# Fish Skin Acellular Dermal Matrix Combined With Negative Pressure Wound Therapy for Diabetic Foot Ulcers

**DOI:** 10.7759/cureus.80488

**Published:** 2025-03-12

**Authors:** Audrey Poh Poh Tan, Jack Kian Chng

**Affiliations:** 1 Vascular Surgery, Singapore General Hospital, Singapore, SGP

**Keywords:** diabetic, fish skin, negative pressure, ulcer, vascular, wound

## Abstract

Background

Managing diabetic foot ulcers (DFUs) is challenging due to poor blood supply, which leads to chronic wounds, increased susceptibility to infections, ischemia, and necrosis. The compromised quality of surrounding tissues, combined with complex underlying conditions and weakened immune systems, often hinders proper wound healing. Fish skin acellular dermal matrix (ADM) has emerged as an effective treatment option to promote healing in such wounds.

Objective

This study is the first to explore the use of fish skin ADM combined with negative pressure wound therapy (NPWT) for treating DFUs in the Asian population. The aim is to demonstrate the efficacy and effectiveness of this combined treatment approach.

Methods

Six patients with DFUs who visited the vascular surgery department between November 2022 and June 2023 were included in the study. Their wounds were treated with Kerecis^®^ Omega3 Wound dressing for definitive closure, while NPWT was applied as an adjunct therapy to enhance graft uptake when suitable.

Results

The average initial wound size was 34.30 cm², with a complete healing rate of 100% achieved over an average of 19 weeks using Kerecis^®^ Omega3 Wound dressing and adjunct NPWT. By the 12-week mark, the average reduction in wound size was 80.50%.

Conclusions

Fish skin ADM offers a biocompatible and sustainable solution for improving wound healing in difficult-to-treat DFUs. The addition of NPWT appears to enhance graft uptake, shorten the time to complete wound closure, and lower the risk of infections commonly associated with chronic diabetic foot wounds.

## Introduction

Diabetic foot ulcers (DFUs) present significant challenges in management due to their close association with the vascular system. These wounds commonly result from poorly controlled diabetes accompanied by peripheral neuropathy and peripheral arterial disease. The primary complications arise from impaired blood supply, which triggers a series of detrimental effects, including delayed wound healing, wound chronicity, heightened susceptibility to infections, and potential progression to ischemia and necrosis [1,2].

The compromised quality of surrounding tissues, along with complex underlying conditions and weakened immune systems, often prevents proper wound closure, resulting in chronic, nonhealing wounds with a high risk of recurrence. Due to the prevalence of DFUs and the limited available treatment options, there has been a strong push to explore new and improved management strategies.

One promising innovation gaining attention is the use of fish skin acellular dermal matrix (ADM). Kerecis^®^ Omega3 Wound, produced by Kerecis hf, Isafjördur, Iceland, is made from decellularized and sterilized North Atlantic cod skin. Previous studies have demonstrated its effectiveness in treating various wound types, particularly diabetic and venous leg ulcers [2-5].

Fish skin ADM is rich in omega-3 fatty acids, which promote healing and tissue repair. These fish skin-based products provide a biocompatible and natural scaffold that supports cellular growth and regeneration of damaged tissues, thereby facilitating the wound healing process [5,6].

In this retrospective case series and literature review, we aimed to evaluate the efficacy of Kerecis^®^ dressing combined with negative pressure wound therapy (NPWT) as an adjunctive treatment for DFUs. This study represents the largest case series to date describing the combined use of fish skin ADM and NPWT for DFU management and is the first to report its use in the Asian population.

## Materials and methods

**Figure 1:**
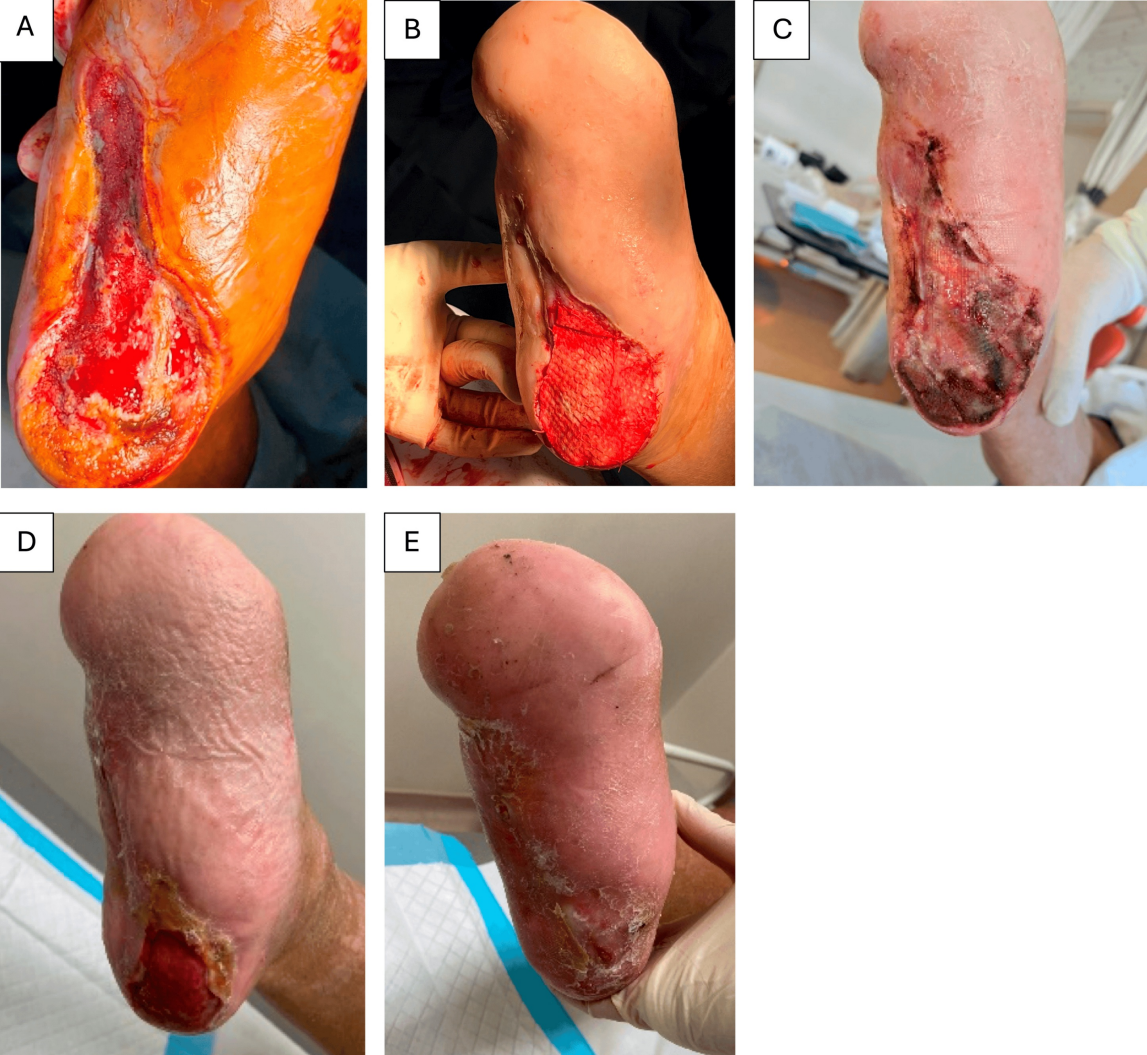
Case 2: A 54-year-old male with a DFU progressing to a large 66 cm² right heel wound underwent (A) repeated wound debridement, (B) followed by a second Kerecis® application 12 weeks after the initial treatment. (C) Wound inspection on postoperative day 3 and (D) four weeks postoperatively showed increased incorporation of fish skin ADM, after which adjunct NPWT was initiated. (E) A review at 20 weeks postoperatively (a total of 32 weeks after the initial Kerecis® application) demonstrated definitive wound closure achieved through the combined treatment. ADM, acellular dermal matrix; DFU, diabetic foot ulcer; NPWT, negative pressure wound therapy

**Figure 2:**
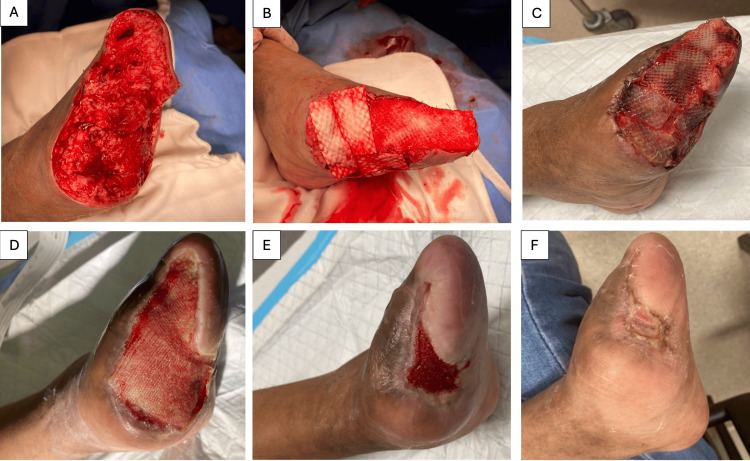
Case 3: A 37-year-old female with a DFU progressing to gangrene of the left first to three toes and dusky appearance of the fourth and fifth toes eventually underwent (A) left forefoot amputation followed by (B) Kerecis® application. (C) Wound inspection on postoperative day 3 and (D) four weeks postoperatively showed increasing incorporation of fish skin ADM, after which adjunct NPWT was initiated. (E) A 10-week postoperative review and (F) a 12-week postoperative review demonstrated definitive wound closure achieved through the combined treatment. ADM, acellular dermal matrix; DFU, diabetic foot ulcer; NPWT, negative pressure wound therapy

**Figure 3:**
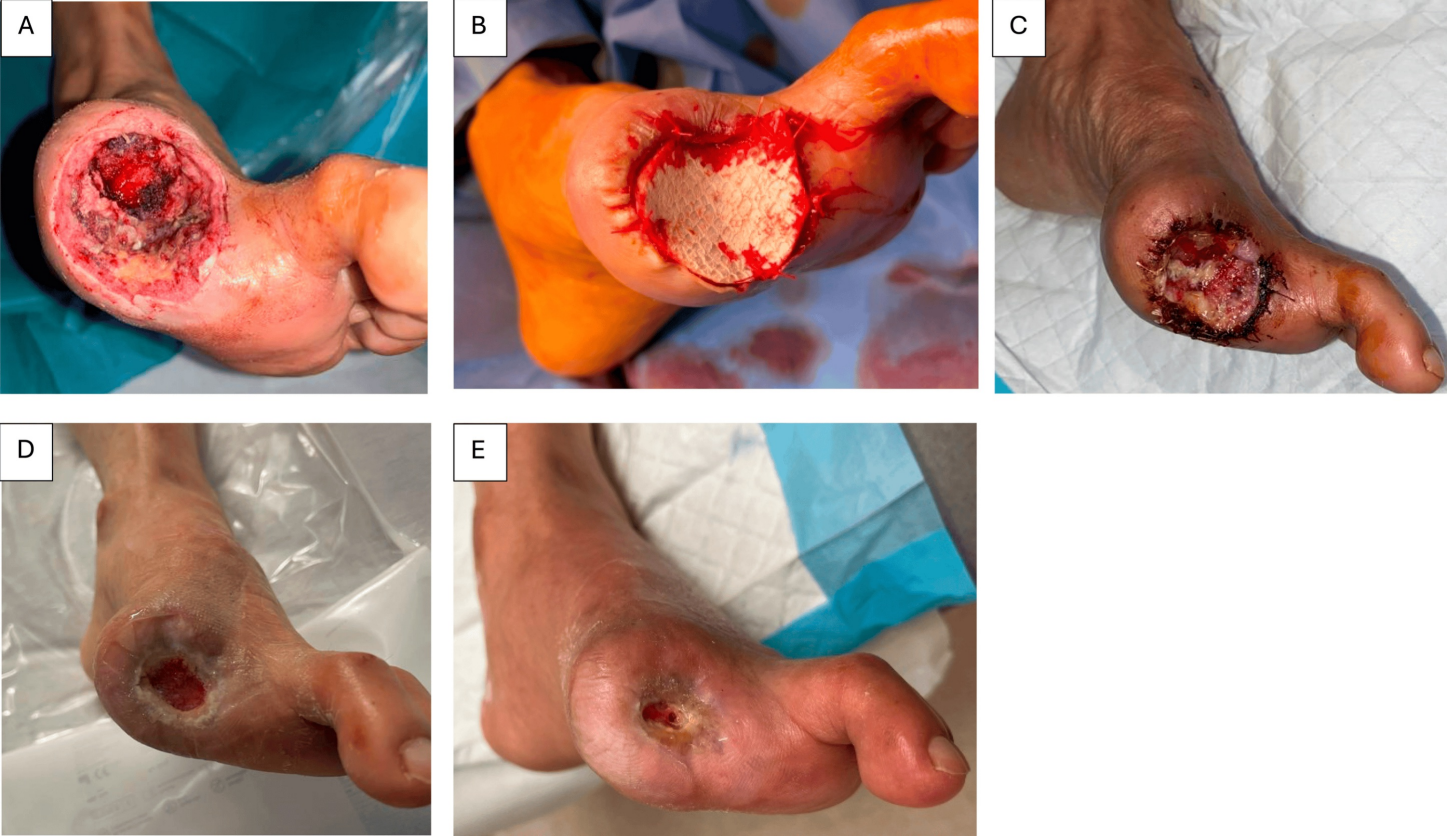
Case 4: A 65-year-old male with a DFU progressing to gangrene of the left big toe underwent (A) left big toe ray amputation followed by (B) Kerecis® application. (C) Wound inspection on postoperative day 7 and (D) 10 weeks postoperatively showed increasing incorporation of fish skin ADM, after which adjunct NPWT was initiated. (E) A 12-week postoperative review demonstrated near-complete wound closure achieved through the combined treatment, with complete healing achieved 16 weeks after Kerecis® application. ADM, acellular dermal matrix; DFU, diabetic foot ulcer; NPWT, negative pressure wound therapy

A total of six patients with DFUs who presented to the vascular surgery department at a tertiary hospital in Singapore between November 2022 and June 2023 were recruited for this study. Table 1 provides a summary of the patients’ details and the progress of their wound management. Additionally, Figure 1, Figure 2, and Figure 3 illustrate the wound healing progression for Case 2, Case 3, and Case 4, respectively.

**Table 1:** Summary of patients recruited for Kerecis® Omega3 Wound application combined with adjunct NPWT for managing DFUs DFU, diabetic foot ulcer; DM, diabetes mellitus; ESRF, end-stage renal failure; HTN, hypertension; HLD, hyperlipidemia; IHD, ischemic heart disease; MCA, middle cerebral artery; NASH, non-alcoholic steatohepatitis; NPWT, negative pressure wound therapy; OSA, obstructive sleep apnea; PVD, peripheral vascular disease; WPW, Wolff-Parkinson-White syndrome

Case	Age (years)	Gender	DM	PVD	Other comorbidities	Wound location	Initial wound size	Wound size at 12 weeks follow-up	Wound size reduction at 12 weeks (%)	Complete wound healing and duration (if yes)
1	59	Female	Yes	No	Nil	Right heel	3.8 × 2.5 cm (9.5 cm²)	Wound completely healed	100%	Yes; 12 weeks
2	54	Male	Yes	Yes	IHD, HTN, HLD, and diabetic nephropathy	Right heel	11.0 × 6.0 cm (66.0 cm²)	7.5 × 5.2 cm (39.0 cm²); required second wound debridement and Kerecis^®^ application	41%	Yes; 32 weeks
3	37	Female	Yes	Yes	ESRF, mixed anemia, and HTN	Left forefoot amputation	7.1 × 6.3 cm (44.7 cm²)	Wound completely healed	100%	Yes; 12 weeks
4	65	Male	Yes	Yes	Previous left high cortical MCA territory infarct and IHD	Left first toe ray amputation	3.0 × 2.8 cm (8.4 cm²)	1.0 × 0.5 cm (0.5 cm²)	94%	Yes; 16 weeks
5	44	Male	Yes	Yes	ESRF, HTN, HLD, IHD, obesity, NASH liver cirrhosis, OSA, chronic lymphedema with elephantiasis, WPW s/p multiple pathway ablations, and sinus node dysfunction s/p permanent pacemaker insertion	Left forefoot amputation	9.0 × 6.0 cm (54 cm²)	6.0 × 3.0 cm (18 cm²)	67%	Yes; 24 weeks
6	67	Female	Yes	Yes	ESRF, coronary artery disease, HTN, HLD, and atrial fibrillation	Left first toe ray amputation	6.1 × 3.8 cm (23.2 cm²)	2.8 × 1.6 cm (4.5 cm²)	81%	Yes; 18 weeks

Before initiating wound management with fish skin ADM, all patients were assessed for peripheral vascular disease and treated with revascularization of the affected vessels. Successful revascularization was confirmed through digital subtraction angiography. Proper wound debridement and sepsis control, with clear signs of wound granulation, were ensured before applying fish skin ADM. While patients were routinely offered split-thickness skin grafting for further wound management, they declined and opted for fish skin ADM as an alternative treatment.

Wounds were managed using Kerecis^®^ Omega3 Wound dressing for definitive closure, with NPWT applied as adjunctive therapy when feasible to enhance graft uptake. Before application, Kerecis^®^ Omega3 Wound dressing was rehydrated with standard saline solution, then applied directly to the wound, secured with sutures, and covered with gauze and crepe bandage.

Inclusion criteria for adjunct NPWT were as follows: (i) successful uptake of Kerecis^®^ dressing; (ii) suitable wound location for NPWT application; and (iii) a healthy wound base. Regular wound inspections were conducted to monitor graft uptake. Once the matrix was well incorporated and surrounding tissue showed no signs of recurrent infection, NPWT was initiated. The timing of NPWT application was based on previous studies indicating that NPWT promotes wound healing in areas without localized ischemia, necrotic tissue, infection, or exposed tendons, nerves, or vessels [7].

This study received Institutional Review Board (IRB) approval under CIRB 2024-2226. Kerecis^®^ Omega3 Wound has obtained FDA authorization and European regulatory approval for use in the United States, Europe, and several Asian countries, including Singapore. Informed consent was obtained from all patients according to institutional protocols, with thorough counseling provided before any wound management procedures.

A comprehensive literature search was conducted using the PubMed database for studies published up to March 31, 2024. The search terms included “kerecis”, “fish skin”, “diabetic”, “foot”, “wound”, and “ulcer,” without applying filters or restrictions. The inclusion criteria were as follows: (i) any study or case report; (ii) studies involving fish skin dressing; and (iii) DFUs. Studies not involving human subjects or not written in English were excluded, resulting in the identification of 14 relevant studies.

A second literature search focused on the use of fish skin ADM combined with NPWT as adjunctive therapy. The keywords used were “kerecis”, “fish skin”, “negative pressure”, “vacuum”, “diabetic”, “wound”, and “ulcer”. No relevant studies addressing chronic DFUs were found.

A third search targeted wound management of DFUs in end-stage renal failure patients, using keywords such as “negative pressure”, “vacuum”, “renal failure”, “diabetic”, “wound”, and “ulcer”. Only one relevant study was identified - a retrospective cohort study evaluating NPWT efficacy in high-risk patients with multiple comorbidities. The study found that patients receiving NPWT healed faster despite having significant comorbidities; however, the exact reduction in healing time was not quantified [8].

## Results

Six patients with DFUs were managed using fish skin ADM and adjunct NPWT, with follow-ups conducted over at least three months, as detailed in Table 1. The patients ranged in age from 37 to 67 years, with an equal distribution of genders. Five of the six patients had both diabetes mellitus (DM) and peripheral vascular disease, while only one had a pure DFU. Notably, five of the patients had significant comorbidities, including ischemic heart disease, chronic kidney disease, and end-stage renal failure, with three specifically having end-stage renal failure.

A successful outcome of Kerecis^®^ dressing application was defined as definitive wound closure, while treatment failure was characterized by recurrent wound infection requiring further amputation or reverting to conventional wound dressing.

The average initial wound size was 34.30 cm², with a complete healing rate of 100% achieved over an average duration of 19 weeks. The average reduction in wound size at the 12-week mark after Kerecis^®^ application with adjunct NPWT was 80.50%. All patients required only a single application of Kerecis^®^, except for Case 2, as shown in Table 1. None of the patients required additional surgical debridement or interventions such as higher-level amputation.

## Discussion

DM is a global epidemic affecting over 420 million people and continues to rise. Singapore is particularly impacted, with one in three individuals at risk of developing DM during their lifetime. Patients with DM have a 19-34% lifetime risk of developing DFUs [9], and those with DFUs face at least a 19% lifetime risk of lower-extremity amputation [10]. Chronic, unresponsive diabetic foot wounds are highly susceptible to infection and often require lower-extremity amputation. Therefore, prompt wound healing is essential to minimize the risk of infection.

A 2020 systematic review reported five-year re-amputation rates of up to 60.7% and five-year mortality rates of up to 74.1% among DFU patients who had previously undergone lower extremity amputations [11]. Beyond the severe psychological, emotional, and quality-of-life impacts, the economic burden of managing these chronic wounds remains substantial. As a result, advanced therapies are critical to improving healing rates, patient outcomes, cost efficiency, and reducing the risk of amputation [2].

An ideal wound dressing offers a moist environment, effective antimicrobial protection, and inhibits metalloproteinase proliferation at a reasonable cost. Studies have demonstrated the effectiveness of collagen matrices for wound management, derived from sources such as porcine, bovine, and more recently, piscine origins.

Numerous studies have established the efficacy and superiority of fish skin ADM over standard collagen alginate dressings for treating chronic DFUs [1-4,12-16]. Lullove et al. conducted a multicenter randomized controlled trial comparing acellular fish grafts to standard collagen alginate dressings (Fibracol Plus Collagen Wound Dressing with Alginate, 3M, St. Paul, Minnesota, USA) for managing chronic nonhealing DFUs. The fish skin ADM group achieved a significantly higher healing rate of 56.9% compared to 31.4% with the standard of care at 12 weeks [2-4]. The average wound size was 3.9 cm² in the fish skin ADM group versus 4.9 cm² in the control group, with an average of 5.9 applications of fish skin ADM. The mean healing time for both groups was seven weeks. However, patients were excluded from the study if their ulcers did not achieve at least a 50% reduction in wound area at the six-week mark, and these results were omitted [4].

Studies evaluating fish skin ADM in chronic DFUs have reported average healing times ranging from seven to 23 weeks, typically requiring multiple applications of fish skin ADM, averaging between 4.5 and 26 applications per wound [1-4,6,13,16]. Faster healing times were generally associated with smaller initial wound sizes. Baldursson et al. conducted a double-blind, randomized controlled trial comparing fish skin ADM to porcine extracellular matrix and found significantly faster healing rates with fish skin ADM (77.5% vs. 65% healed wounds at 25 days) [17]. Yoon et al. and Stone et al. also demonstrated quicker healing with fish skin ADM compared to commonly used bovine collagen skin grafts for burn patients [18,19]. Additionally, fish skin ADM exhibited more rapid integration, accelerated wound re-epithelialization, contraction, and overall wound closure compared to bovine grafts [18].

Fish skin ADM has gained increasing attention due to concerns associated with mammalian-derived ADMs, such as the risk of autoimmune responses [20], prion disease [21], and cultural restrictions against using bovine and porcine products in certain countries. Piscine grafts offer a compelling alternative, addressing these issues while providing additional benefits from omega-3 polyunsaturated fatty acids. Unlike mammalian grafts, which require harsh detergents during sterilization to prevent viral transmission - resulting in unintended stripping of fat cells - fish skin ADM undergoes gentler processing.

Studies have shown that wounds treated with fish skin ADM do not induce seroconversion of autoantibodies [17], and there is no known risk of viral transmission from North Atlantic cod to humans. Fish skin’s molecular structure closely resembles that of mammalian skin, making it a suitable substitute [22]. Its processing retains essential structural and molecular components, including collagen, fibrin, proteoglycans, glycosaminoglycans, and growth factors [13]. The resulting product is a lyophilized acellular fish skin with a porous, homologous scaffold that is non-allogenic to humans, with a particularly distinctive feature: intact bioactive lipid mediators [2].

This porous structure promotes superior three-dimensional wound cell ingrowth, even outperforming human allografts [23]. Bioactive lipid mediators like omega-3 polyunsaturated fatty acids, eicosapentaenoic acid, and docosahexaenoic acid contribute to the anti-inflammatory and anti-infective properties of fish skin ADM. They achieve this by enhancing antibacterial and antiviral activity [22] and inhibiting the secretion of the pro-inflammatory cytokine interleukin 1-beta by macrophages [24].

Chronic wounds often stall in the inflammatory phase and fail to progress through the healing process. The bioactive pro-resolving lipid mediators present in Kerecis^®^ promote the resolution of this chronic inflammatory state, facilitating the wound healing process.

NPWT has proven effective in reducing the complexity and size of diabetic foot wounds and promoting deep wound healing [25]. Negative pressure exerts micro-strain, mechanically stretching cells to encourage rapid division, proliferation, and growth factor production essential for wound healing [26]. NPWT also assists in removing exudate and bacteria, improving wound bed oxygenation, and increasing skin graft uptake from 72% to 92% [27]. Other factors influencing successful graft uptake include fluid elimination, graft immobilization, securement, and stabilization on irregular surfaces [28], all of which are addressed by NPWT.

To date, only two studies have examined the combined use of fish skin ADM and NPWT. One case series demonstrated this approach in acute pediatric wounds [29], while another highlighted a case involving combat injuries from a military drone assault [30]. However, no studies have specifically investigated the combined use of NPWT and fish skin ADM in managing DFUs.

Our study demonstrates the novel application of fish skin ADM and NPWT for treating diabetic foot wounds. The overall complete healing rate was 100% over an average duration of 19 weeks with Kerecis^®^ Omega3 Wound dressing, which aligns well with previously reported results. Among patients who achieved definitive wound closure, only one required a second Kerecis^® ^application, while the others healed successfully with a single application. The need for a second application was likely due to the large wound size of 66 cm² and the challenging nature of the wound, which showed only a 41% reduction at the 12-week mark.

Our center’s experience with fish skin ADM and NPWT suggests several factors contribute to successful wound closure, including initial wound size and patient comorbidities. Fish skin ADM presents a valuable alternative for patients unwilling to undergo split skin grafting, providing effective wound healing without the associated risks and complications. The adjunct use of NPWT highlights its benefits, including fewer applications, reduced interventions, and prevention of disease progression that could necessitate higher amputations.

This study’s limitations include the small sample size and relatively short follow-up period, which may affect the representativeness of the findings. As a retrospective case series without a control group, confounding factors may be present. Larger randomized controlled studies are needed to further substantiate the efficacy of fish skin ADM when used alongside NPWT.

## Conclusions

Fish skin ADM offers a biocompatible and sustainable approach to enhancing wound healing for difficult-to-treat chronic wounds like DFUs. The use of NPWT has shown potential in promoting graft uptake, accelerating wound closure, and reducing the risk of infections associated with chronic diabetic foot wounds. These findings suggest that combining fish skin ADM with NPWT could be a valuable treatment strategy. However, further large-scale randomized controlled studies are needed to thoroughly evaluate the effectiveness of this combined approach and establish standardized treatment protocols.
